# Public speaking anxiety decreases within repeated virtual reality training sessions

**DOI:** 10.1371/journal.pone.0216288

**Published:** 2019-05-31

**Authors:** Marcel Takac, James Collett, Kristopher J. Blom, Russell Conduit, Imogen Rehm, Alexander De Foe

**Affiliations:** 1 RMIT University, Melbourne, Australia; 2 Virtual Human Technologies, Vinohrady, Czech Republic; University rehabilitation institute, SLOVENIA

## Abstract

Therapy for public speaking phobia using virtual reality exposure (VRE) has focused on distress arousal rather than distress habituation. Understanding habituation will help optimise session duration, making treatment more affordable and accessible. This pilot study utilised within-speech repeated measures to examine distress habituation during three brief public speaking scenarios in a non-clinical sample (*n* = 19; 18–76 years). VRE elicited significant distress in all three scenarios. Although within-scenario distress habituation was not observed, between-scenario habituation was partially supported. An increase in distress during the second scenario indicated that three consecutive speech performances were critical in achieving habituation. Brief repeated VRE scenarios using an agent audience were effective in eliciting public speaking distress, as well as habituation.

## Introduction

Fear of public speaking (FOPS) is associated with debilitating anxiety that impacts social, academic, and career opportunities [1]. While cognitive-behavioural therapy (CBT) with graduated exposure is an effective intervention [2], in vivo exposure requires a suitable venue and audience. This complicates treatment and increases cost. Despite evidence supporting virtual reality exposure (VRE) as a suitable treatment alternative [3], research limitations are evident. Phobia research has generally focused on ensuring that VRE elicits distress [4], however there is a need to also examine distress *habituation*. Improved understanding of habituation patterns will promote optimisation of FOPS detection, intervention and prevention, and may also contribute to improved therapy access [5].

### Fear of public speaking

FOPS is classified as a non-generalised social anxiety disorder (SAD) associated with performance situations involving perceived scrutiny by others [6]. The age of onset for social anxiety is typically 8–15 years [6] with FOPS being the most common lifetime social fear (21.2%) [7]. When facing public speaking, individuals with FOPS overestimate the potential for negative evaluation by others [8] while underestimating their own capability [9]. Thus, public speaking situations can become highly unpleasant and personally threatening [9].

FOPS includes both physiological (e.g., shaking) and psychological (worry) symptoms [10]. In some case, public speaking anxiety can culminate into panic attacks [11]. In addition, physiological FOPS symptoms can be visible to others, thus contributing to performance anxiety and discomfort. While individuals with FOPS anxiety try to avoid public speaking scenarios [6], avoidance actually limits their ability to overcome fear through experience [9]. Hence, FOPS limits functioning and lifestyle through fear-related academic and career avoidance [12, 13]. Empirically validated interventions are necessary for alleviating these detrimental outcomes of FOPS.

### Integrating virtual reality into public speaking therapy

The most empirically supported FOPS intervention is CBT [2]. Individual and/or group CBT is an effective FOPS treatment, with long-term success reported [14, 15]. Controlled exposure allows individuals to experience public speaking, despite feeling some anxiety [2]. However, exposure requires coordination of venues and suitable audiences, which can delay therapy and increase costs.

A newer approach to FOPS treatment utilises virtual reality (VR) to emulate FOPS scenarios [16]. VRE can be delivered on demand, through a commercially available head-mounted display [17]. Such VR displays incorporate audio, as well as head-tracking, which maintains a user-centered field-of-view [16]. However, VR is more than technology; it is a psychological experience that creates the perception of being physically present in a virtual environment [18]. This sense of presence is considered to heighten emotional responses, such as anxiety [19].

VR delivers consistent and predictable public speaking scenarios that are highly customisable. This allows for systematic gradation of the challenge level of public speaking scenarios [20]. Empirical evidence supports VRE efficacy for FOPS [8, 21]. Anderson, Price [22] determined that VRE and in vivo exposure demonstrated similar positive FOPS outcomes in a SAD-diagnosed sample (*n* = 97). VRE altered real-world behaviour, with a reduction in peak anxiety reported. A 63% rate of partial or full remission of original diagnosis was achieved after three months [22]. Follow-up at 6-years indicated comparable long-term benefits for both treatments [3].

### Distress habituation

Distress habituation is an indicator of emotion regulation and treatment efficacy [23], and is operationalised as a significant reduction in distress during and between exposures [24]. Although detailed FOPS habituation literature is lacking, the value of habituation research is evident in other domains. For example, in a 10-week clinical post-traumatic stress disorder (PTSD) study, van Minnen and Foa [24] compared 30-minute (*n* = 32) with 60-minute (*n* = 60) imaginal exposure and determined that while within-session habituation differed, between-session habituation was equally effective. Van Minnen and Foa [24] reported a significant reduction in PTSD symptom severity between weeks 2–10, for both groups.

Thus, habituation-based evidence allowed a reduction in therapy session duration without compromising therapy efficacy [24]. Shorter sessions reduce cost and make therapists more accessible [24]; a desired outcome for FOPS interventions [5]. Mapping FOPS symptomology against VRE habituation may also identify what symptoms or individual characteristics delay treatment response [24]. Finally, mapping within-scenario distress and habituation patterns of clinical and non-clinical populations could improve FOPS clinical diagnosis by providing reference data.

### Previous research Findings and limitations

Based on Foa and Kozak’s [23] emotional processing theory, research by Finn, Sawyer [25] provides an empirical foundation for public speaking distress habituation. In a quasi-experimental design (*n* = 140), Finn, Sawyer [25] assessed anxiety following two 5-minute speeches presented to class peers: 1. start-of-semester, and 2. end-of-semester. Treatment exposure consisted of brief repeated in vivo speeches to class peers, delivered during the semester. While within-scenario distress habituation for both speeches occurred independent of treatment, a significant between-scenario interaction of time by group, and a main effect for time were observed [25]. The experimental group experienced significantly less anxiety than controls during the end of semester speech; an outcome considered to be the result of experimental exposure [25].

As a limitation, Finn, Sawyer [25] suggested that audience familiarity should be controlled in future designs; a logistical hurdle for in vivo exposure. Furthermore, anxiety analysis was limited to the two, start and end-of-semester speeches (i.e., no assessment during brief exposure treatment). Finally, distress data collection occurred post-speech. While post-speech measures are common within public speaking literature, this methodology requires participants to reflect on their experience, which could make subjective measures less accurate.

Although within-speech habituation literature is lacking, preliminary VRE research has examined between-session habituation. Harris, Kemmerling [26] compared brief VRE FOPS exposure (*n* = 8) with waitlist (*n* = 6); measures included the Personal Report of Confidence as a Speaker (PRCS) [27], Subjective Units of Discomfort Scale (SUDS) [28] and heart rate. Weekly VRE scenarios were designed to provide increasing situational difficulty. While the treatment group results indicated a significant reduction in PRCS scores and heart rate, no significant SUDS decrease was observed. Harris, Kemmerling [26] suggested that the lack of SUDS reduction, despite increasing scenario difficulty, may indicate improved distress tolerance (i.e., habituation). Kozasa and Leite [29] also applied this rationale. However, the Kozasa and Leite [29], and Harris, Kemmerling [26] studies did not randomise scenarios to assess the assumption of anxiety stability despite increased task difficulty. Therefore, a randomised assessment is warranted.

Given the potential suitability of VRE to employ repeated measures in randomised scenarios, such methodology has not been explored within VRE public speaking literature. This could be partly attributed to the VRE public speaking research focus on eliciting anxiety and presence; methodologies that do not utilise within-speech measures. For example, Owens and Beidel [30] assessed VRE and in vivo differences for SUDS, heart rate and presence in adults with and without SAD; limited to baseline and post-speech measures. A significant increase in heart rate was evident for VRE and in vivo, with no group differences observed [30]. VRE and in vivo elicited significant SUDS increases (over baseline), with in vivo distress being significantly higher than VRE when in vivo was the second scenario [30]. However, presence-focused VRE literature does not consider within-speech distress variability (i.e., habituation), which could differ for in vivo and VRE. Thus, within-speech measures during VRE should be assessed.

### The present study

This pilot study employed new research methodology, specifically focused on VRE-specific capabilities to overcome gaps within public speaking literature. Firstly, to address the existing dependence on post-speech anxiety measures, the present study utilised a repeated measures design to assess within-speech distress and habituation. In addition, to assess the assumption that anxiety score consistency (despite increased scenario difficulty) represents habituation, three agent-based VRE public speaking scenarios of varied difficulty were randomised, along with three impromptu speech topics in a single 60-minute session. This repeated randomised design was introduced to control for graded scenario difficulty across the three speeches. A non-clinical sample was utilised to gauge (sensitivity of) emotional regulation within public speaking VRE environments. The rationale was that a non-clinical sample would have lower sensitivity to FOPS, which would facilitate a better assessment of software capability (i.e., agent-based distress would be harder to elicit with a non-clinical sample).

The first aim was to gauge VRE effectiveness of agent-based software in initiating public speaking distress during brief repeated exposures. It was hypothesised that public speaking VRE would elicit distress within each scenario. The second aim was to determine whether within-scenario and between-scenario distress habituation was evident through brief repeated VRE. The second hypothesis was that within-scenario habituation would be achieved in each scenario. The third hypothesis was that brief repeated exposure would result in between-scenario habituation.

## Method

### Participants

Participants were recruited through RMIT University, Melbourne, including the RMIT chapter of Toastmasters (*n* = 5), as well as from the general population using Facebook. Toastmasters is an international organisation focused on development of public speaking and leadership skills. Membership of Toastmasters reflects a self-interest in improving public speaking competence for a variety of reasons (e.g., self-confidence or non-native English speakers). Regular group attendance and public speaking participation are not compulsory. As such, Toastmasters members may not necessarily possess greater capability in managing FOPS than the general population.

A total of 22 adults commenced the study, however, one was unable to complete the procedure due to task-related discomfort and was excluded. The remaining 21 participants were screened for clinical levels of social anxiety. Two participants obtained a score > 34 on the Social Interaction Anxiety Scale (SIAS) [31], indicating clinical SAD levels (per [31]) and were excluded from analysis. Decision to exclude clinically SAD participants was based on the rationale that non-clinical participants would be less sensitive to state anxiety. Therefore, agent-based scenario distress would be harder to initiate, providing a better gauge of software capability. The final sample consisted of 19 participants aged 18–76 years (*M* = 35.47, *SD* = 15.46). There were ten males (52.6%) and nine females (47.4%).

Twelve participants (63.1%) were students (10 fulltime; 52.6%) from various disciplines, including five from psychology-related programs. A total of 14 participants (73.7%) were employed (11 part-time; 57.9%), with one participant working in a psychology-related field. Toastmasters participants represented 26.3% of the total sample (*n* = 5). Participation was voluntary, with a small incentive offered (the chance to win one of three $100 store gift cards).

### Materials

VR equipment consisted of a personal computer, Oculus Rift (Oculus VR, Facebook Inc., USA) and Virtual Orator (Virtual Human Technologies Inc., Czech Republic) public speaking software. Audience agents represented a mix of race and gender; agents were randomised in every scenario. Participants experienced themselves in first-person perspective in the scenarios, as per the standard setup of the Oculus Rift visual experience.

Heart rate was recorded in beats per minute (bpm) using a wrist-worn Fitbit Surge (Fitbit Inc.). Fitbit Surge demonstrates heart rate reliability for laboratory experiments that do not involve excessive physical movement (e.g., running), with error rates below 5% when compared to medical devices [32]. Since heart rate is not a pure measure of distress [33], it was recorded as a secondary measure of arousal.

The SIAS [31] is a self-report measure of fear in general social interactions. Responses to 20 items are scored on a 5-point Likert scale. Ratings ranged from 0 (not at all) to 4 (extremely). The scale has demonstrated high internal consistency (α = .94) and test-retest reliability (α = 0.92) [31].

The short-form of the Personal Report of Confidence as a Speaker (PRCS-2) is a FOPS measure [34]. It consists of 12 true or false items, with possible scores ranging from 0 (lowest fear) to 12 (highest fear). The scale has demonstrated internal consistency (α = .85) and validity, reporting a significant correlation with the 30-item version (*r* = 0.81, *p* < .001, *n* = 1194) [34].

SUDS [28] is a single-item self-report distress measure; distress is rated from 0 (no distress) to 100 (extreme distress), with repeated measures used to monitor distress variance over time. SUDS has been validated in anxiety therapy [28] and research [24, 26, 30]. A reception call-bell (i.e., counter bell) was utilised during VRE to indicate that a new SUDS rating was required.

The Igroup Presence Questionnaire (IPQ) [35] measures four presence constructs (general, spatial, involvement and realness). Self-reported responses are rated on a 7-point Likert scale from 0 (lowest) to 6 (highest), with varied anchors across items. The IPQ constructs demonstrate good validity and internal consistency: spatial α = .85, involvement α = .72 and realness α = .79 [36].

### Procedure

Ethics approval was received from the College Human Ethics Advisory Network (Science, Engineering and Health) at RMIT University. Advertising for participants commenced using printed posters and paid Facebook advertisements. Consenting participants were fitted with a heart rate monitor and introduced to SUDS. The first (resting) SUDS and heart rate measures were taken. Participants then completed pre-exposure measures: demographics, SIAS and PRCS-2. All measures other than heart rate and SUDS were completed via Qualtrics. Heart rate and SUDS were recorded manually by the experimenter and matched to Qualtrics data using a non-identifying participant number.

**Fig 1:**
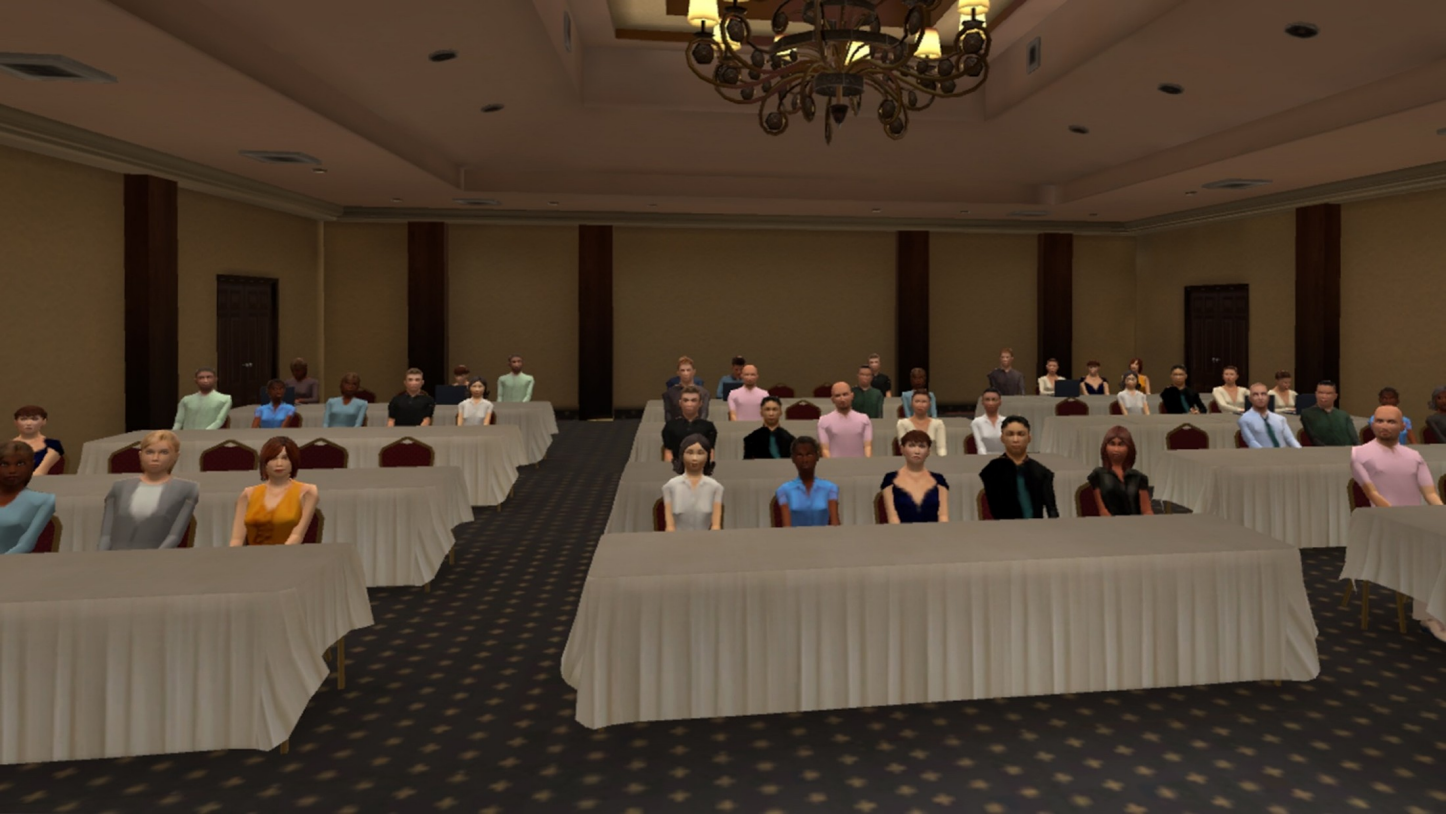
Example of small room scenario.

**Fig 2:**
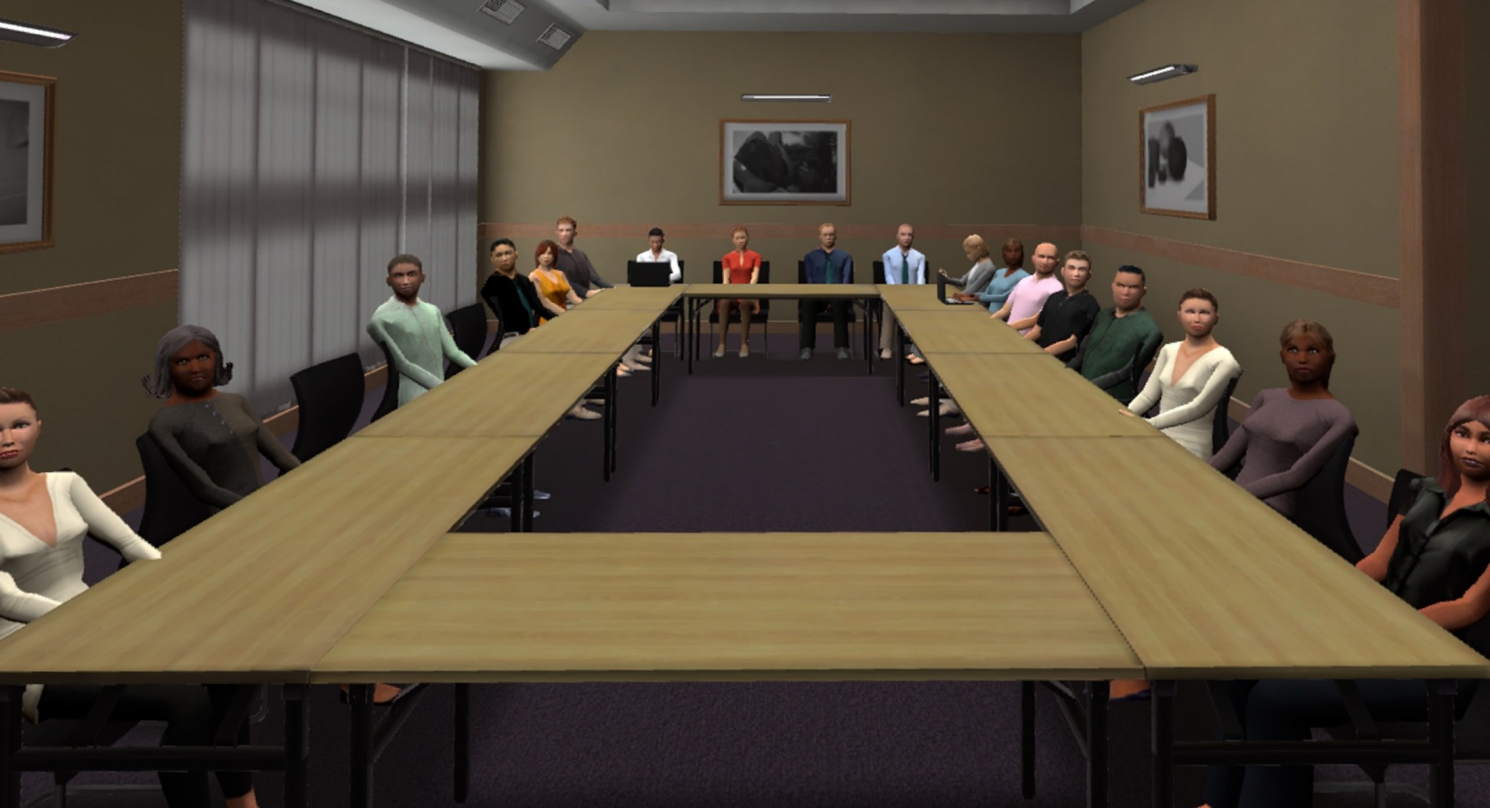
Example of medium room scenario.

**Fig 3:**
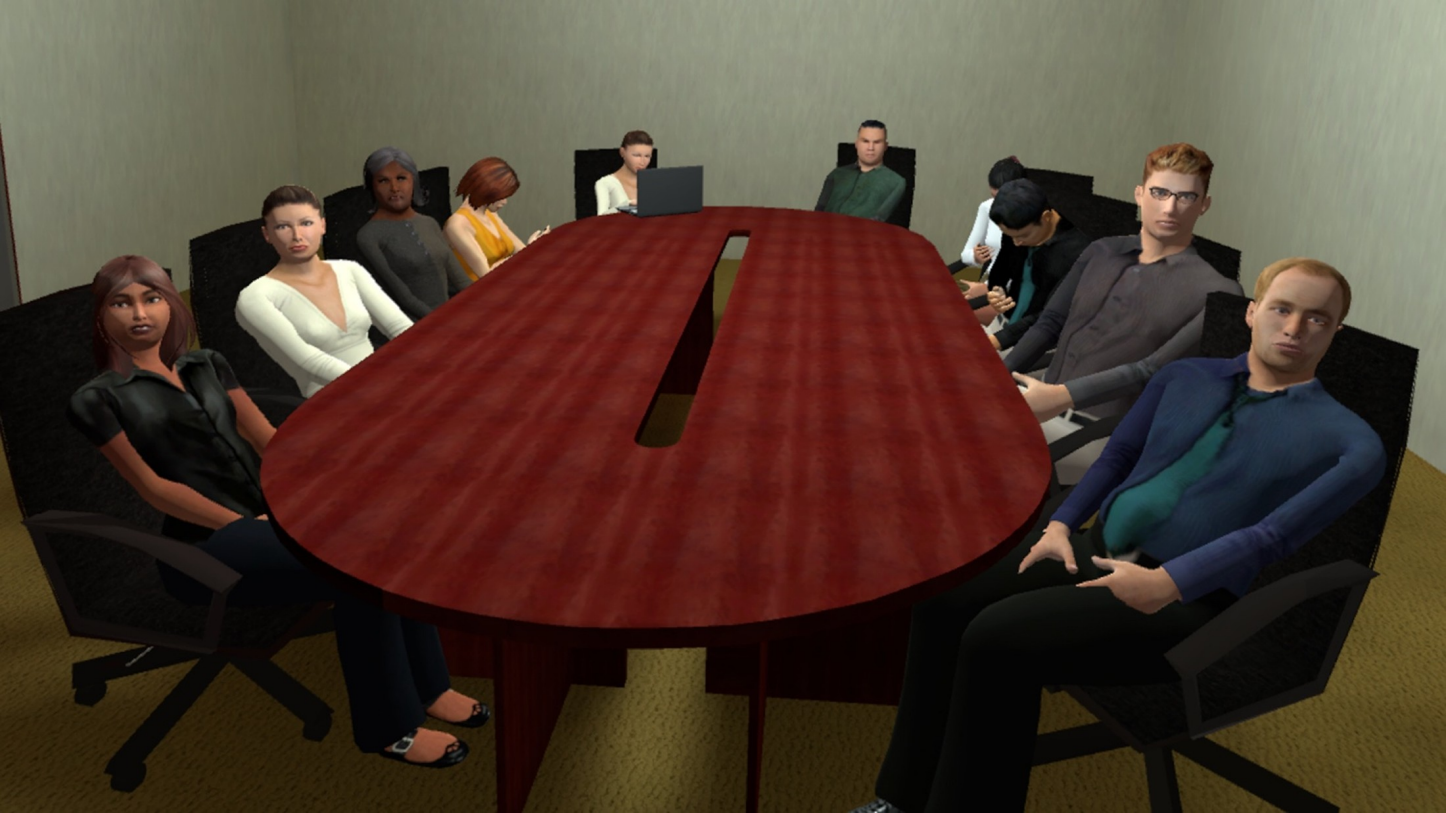
Example of large room scenario.

A VR induction, including a speech-free VRE scenario (audience-free classroom, not used in experiment) was provided. Participants then removed their head mounted display and rested two minutes before receiving their first topic. Three topics and three software scenarios were randomised for each participant. Topics were general knowledge and included descriptions of the audience: 1. provide overview of attractions and character of Melbourne to senior executives, 2. present pros and cons of public transport to a government committee and 3. detail the importance of managing our environment to university students. VRE scenarios consisted of varied venues and audience sizes: small (10 agents; see Fig 1), medium (18 agents; see Fig 2) and large (46 agents; see Fig 3). To increase variety, the small venue audience was programmed to be less attentive to the speaker, with some audience members checking a wall clock and using mobile phones. The medium and large venue audiences were more focused on the presenting participants.

It was requested that participants speak for six minutes, although earlier conclusion was permitted. Participants were given 30-seconds to read each speech topic. Heart rate and SUDS measures were taken again and then three minutes were provided for speech preparation. Participants were able to utilise virtual cue cards if desired. After three minutes of preparation, participants fitted their head mounted display and waited for commencement of the VRE scenario. There was a two-minute pause prior to scenario commencement. Speeches were delivered standing up, as would be common for all the scenarios utilised.

After Speech 1 and 2, the head mounted display was removed and participants sat for three minutes, after which resting heart rate and SUDS were recorded. This process was repeated for the subsequent scenarios.

**Fig 4:**
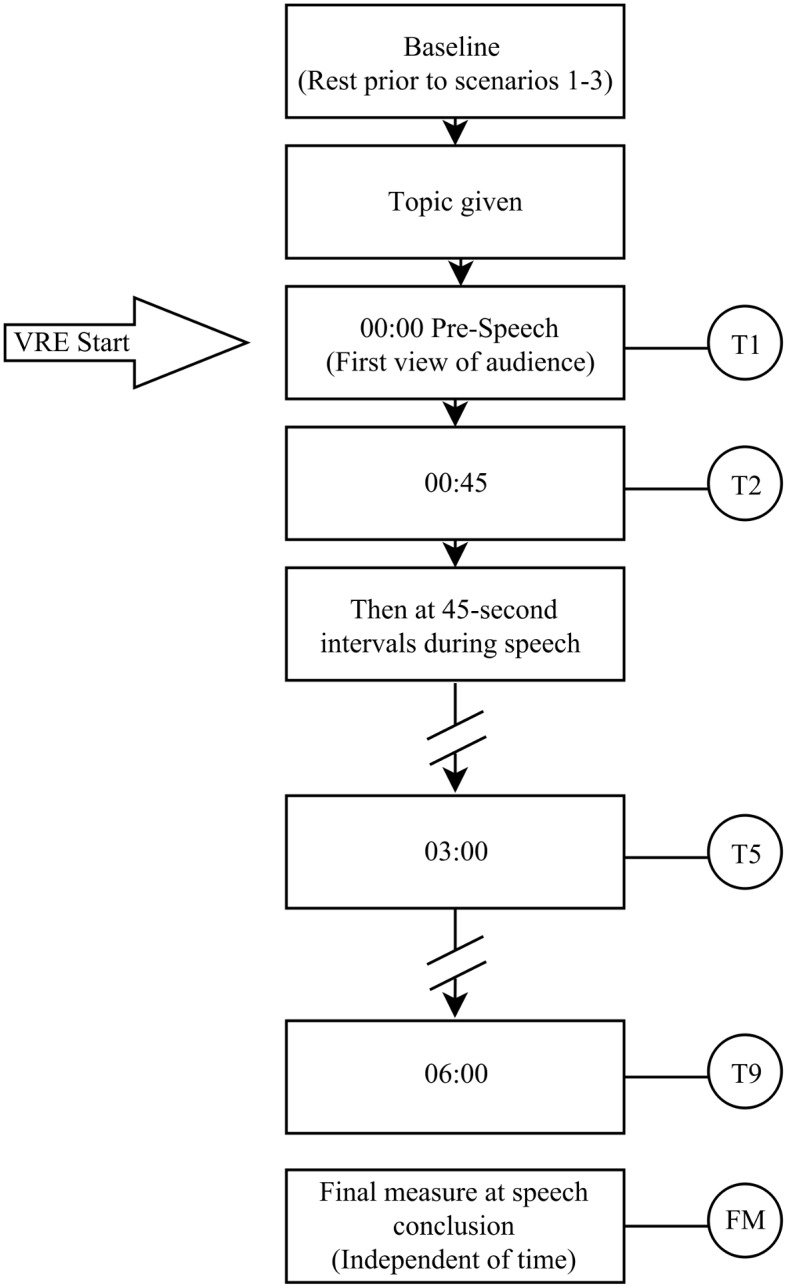
Within-scenario heart rate and Subjective Units of Discomfort Scale measure intervals used.

During VRE scenarios, heart rate and verbal SUDS measures were taken during predetermined milestones, as shown in Fig 4. Following Speech 3, participants completed the IPQ. The whole procedure took approximately 60 minutes.

## Results

### Data preparation and Screening

Self-report measures, heart rate, and SUDS scores were exported to SPSS. Relevant items were reverse coded: SIAS (items 5, 9 and 11) and IPQ (items 3, 9 and 11). Total scores were calculated for SIAS, PRCS-2 and IPQ. When controlling for randomisation, a preliminary t-test showed no significant SUDS or heart rate differences for speech or scenario type, which indicated that each scenario/speech produced a comparable stress response. PRSC-2 data demonstrated a low internal consistency and was therefore excluded from analysis. Descriptive statistics for all self-report measures are presented in Table 1.

**Table 1:** Descriptive statistics for self-report measures (N = 19).

Measure	*M* (*SD*)	Range		Skew	Kurtosis	α
		Actual	Potential			
SIAS	19.79 (7.51)	5–33	0–80	-0.18	-0.53	.82
PRCS-2	5.58 (2.09)	3–10	0–12	0.39	-0.75	.67
IPQ						
Total	46.89 (11.38)	15–61	0–84	-1.58	2.93	.83
General^a^	4.68 (1.46)	1–6	0–6	-1.80	3.08	-
Spatial	20.26 (6.17)	4–27	0–30	-1.25	1.46	.80
Involvement	16.63 (5.15)	7–24	0–24	-0.54	-0.54	.74
Realism	10 (3.01)	3–14	0–24	-0.75	0.11	.11

*Note*. SIAS = Social Interaction Anxiety Scale. PRCS-2 = Short-Form of the Personal Report of Confidence as a Speaker. IPQ = Igroup Presence Questionnaire.

^a^Single scale item.

### Hypothesis-Testing for subjective distress and physiological arousal

Analysis compared mean baseline measures with mean pre-speech (Time 1) measures for SUDS and heart rate for each scenario. For SUDS, histograms indicated one extreme outlier for the first scenario. Calculations were performed with and without the outlier and the pattern of results remained the same. Accordingly, the outlier was retained. Two-tailed, paired samples *t*-tests were performed to compare mean SUDS, and mean heart rate scores across time, with the results shown in Table 2.

**Table 2:** Initial within-scenario Subjective Units of Discomfort Scale and heart rate levels.

Measure	Baseline	T1	*t*	*df*	Effect size	
	*M (SD)*	*M (SD)*			Cohen’s *d*	95% CI
S1						
SUDS	14.47 (12.00)	36.47 (17.85)	6.38**	18	1.45	[0.91, 1.86]
Heart rate	77.21 (9.93)	85.11 (12.16)	3.17**	18	0.71	[0.40, 0.96]
S2						
SUDS	20.32 (13.40)	29.16 (17.40)	2.88**	18	0.57	[0.30, 0.80]
Heart rate	72.42 (8.73)	79.11 (8.72)	4.09**	18	0.77	[0.44, 1.03]
S3						
SUDS	19.53 (11.35)	28.26 (17.01)	3.26**	18	0.60	[0.32, 0.84]
Heart rate	71.47 (9.29)	76.32 (8.12)	4.27**	18	0.56	[0.29, 0.78]

*Note*. SUDS = Subjective Units of Discomfort Scale. S1 = Scenario 1. S2 = Scenario 2. S3 = Scenario 3. Time 1 = 00:00 minutes.

**p < .01, two-tailed.

### Hypothesis-Testing for within-scenario habituation

**Fig 5:**
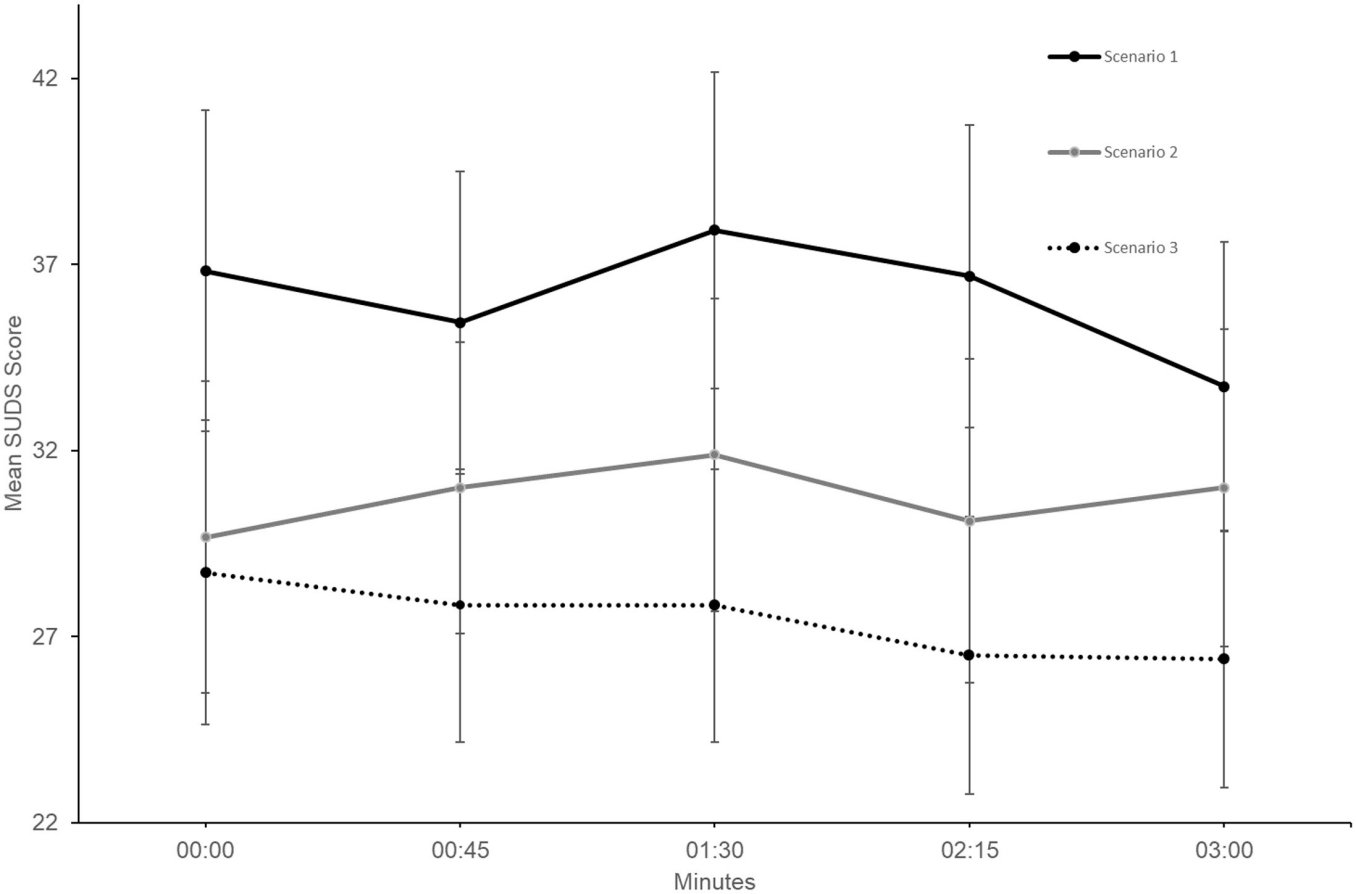
First 3-minutes of within-scenario Subjective Units of Discomfort Scale (SUDS) mean scores for all scenarios (N = 18). Error bars represent standard errors.

Two-tailed, paired samples *t*-tests were used to compare Time 1 measures with time at three minutes (Time 5) measures, for SUDS and heart rate within each scenario. The Time 5 interval was used to maintain a larger sample size as not all participants needed the additional time beyond Time 5. No significant SUDS differences were identified between Time 1 and Time 5 (see Table 3), with an identical result emerging when comparing Time 1 to the participant’s final measure (independent of time). Fig 5 shows SUDS score trends for the first three minutes for all scenarios.

**Table 3:** Paired samples t-test comparisons of mean Subjective Units of Discomfort Scale and heart rate measures.

Measure	Time 1	Time 5	*t*	*df*	Effect size	
	*M(SD)*	*M(SD)*			Cohen’s *d*	95% CI
S1						
SUDS	36.83 (18.29)	33.72 (16.46)	-0.81	17	-0.18	[-0.36, 0.02]
Heart rate	84.33 (12.02)	84.56 (11.06)	0.09	17	0.02	[-0.17, 0.20]
S2						
SUDS	29.67 (17.76)	31.00 (18.08)	0.45	17	0.07	[-0.12, 0.26]
Heart rate	78.78 (8.86)	85.67 (10.57)	2.45*	17	0.71	[0.39, 0.96]
S3						
SUDS	28.72 (17.38)	26.39 (14.60)	-0.85	17	-0.15	[-0.33, 0.05]
Heart rate	76.28 (8.36)	82.06 (10.56)	3.64**	17	0.61	[0.32, 0.84]

*Note*. SUDS = Subjective Units of Discomfort Scale. S1 = Scenario 1. S2 = Scenario 2. S3 = Scenario 3. Time 1 = 00:00 minutes. Time 5 = 03:00 minutes.

*p < .05, two-tailed.

**p < .01, two-tailed.

**Fig 6:**
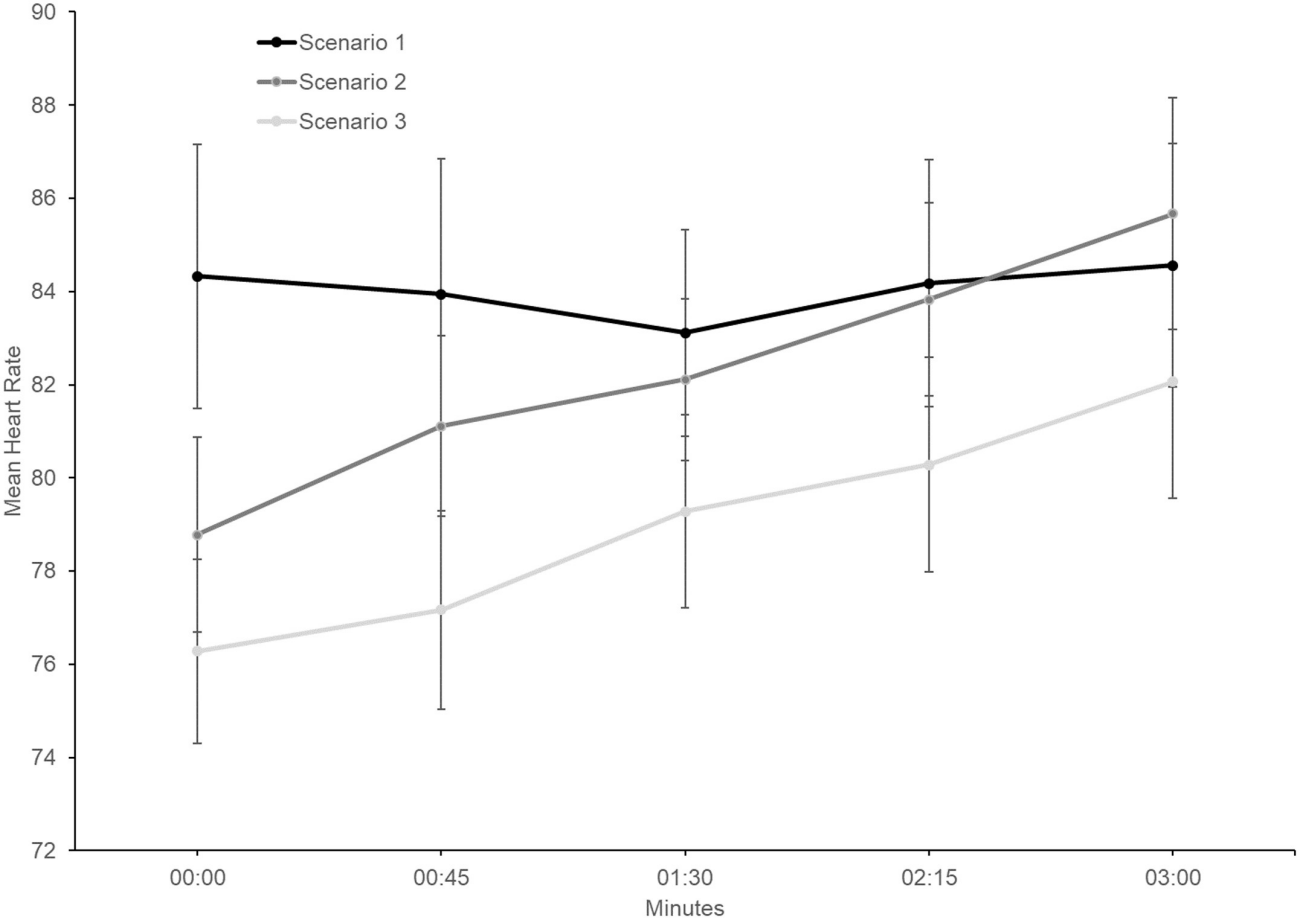
First 3-minutes of within-scenario heart rate mean scores for all scenarios (*N* = 18). Error bars represent standard errors.

Heart rate increases were observed within Scenario 2 and Scenario 3 (Fig 6). Significant between-scenario heart rate differences (from Time 1 to Time 5) were identified for Scenario 2 and Scenario 3 (Table 3).

### Hypothesis-Testing for between-scenario habituation

**Fig 7:**
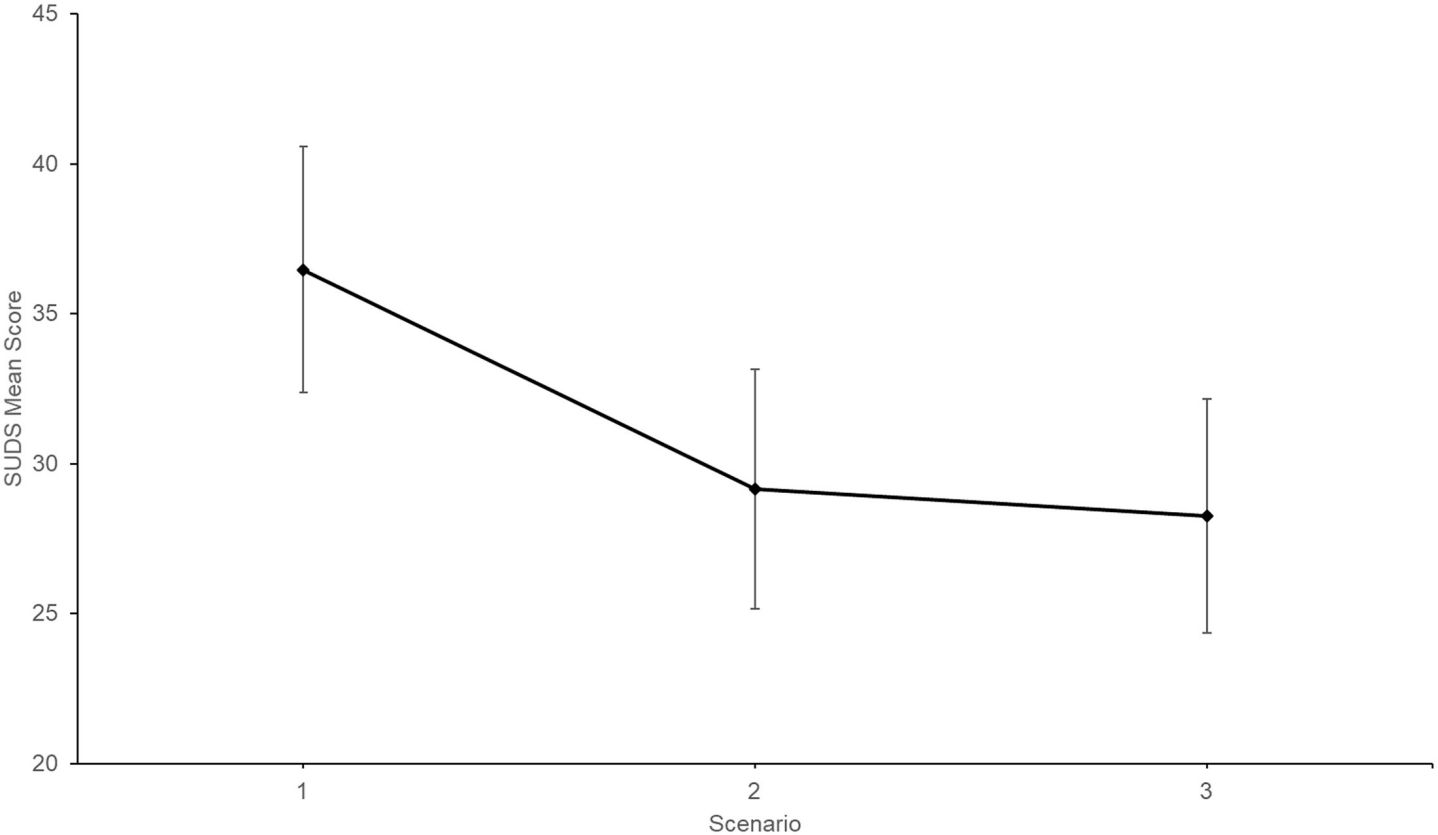
Subjective Units of Discomfort Scale (SUDS) Time 1 mean scores for each scenario. Time 1 = 00:00 minutes. Error bars represent standard errors. (*N* = 19).

One way repeated measures analysis of variance (ANOVA) was performed for SUDS and heart rate using mean Time 1 measures for all scenarios. For SUDS, repeated measures ANOVA results showed that the sample experienced SUDS habituation between some scenarios, *F*(2,36) = 3.997, *p* = .027, partial η^2^ = .18. Fig 7 shows the observed between-scenario SUDS trend.

Given that Bonferroni adjustment detrimentally impacts small samples [37], pairwise comparisons were calculated at the *p* = .05 level (Table 4). Significant differences were observed between Scenario 1 and Scenario 2, as well as Scenario 1 and Scenario 3. No significant difference was observed between Scenario 2 and Scenario 3. A paired samples *t*-test was conducted between Scenario 2 mean SUDS score at Time 5 (*M* = 31.00, *SD* = 18.08) and Scenario 1 mean SUDS score at Time 1 (*M* = 36.83, *SD* = 18.29). The difference was not significant, *t* (17) = 1.33, *p* = .200.

**Table 4:** Pairwise comparisons of mean Subjective Units of Discomfort Scale and heart rate measures (N = 19).

Measures	*M* difference	*SE*	95% CI	Effect size	
				*Cohen’s d*	95% CI
S1 vs. S2					
SUDS	-7.32*	3.10	[-13.82, -0.81]	-0.42	[-0.62, -0.18]
Heart rate	-6.00**	2.03	[-10.26, -1.74]	-0.57	[-0.79, -0.29]
S1 vs. S3					
SUDS	-8.21*	3.76	[-16.12, -0.30]	-0.47	[-0.68, -0.22]
Heart rate	-8.79**	2.08	[-13.15, -4.43]	-0.85	[-1.13, -0.50]
S2 vs. S3					
SUDS	-0.90	2.59	[-6.34, 4.55]	-0.05	[-0.23, 0.13]
Heart rate	-2.79	1.74	[-6.43, 0.86]	-0.33	[-0.52, -0.11]

*Note*. SUDS = Subjective Units of Discomfort Scale. S1 = Scenario 1. S2 = Scenario 2. S3 = Scenario 3. Heart rate = beats per minute.

*p < .05, two-tailed.

**p < .01, two-tailed. Unadjusted for multiple comparisons

**Fig 8:**
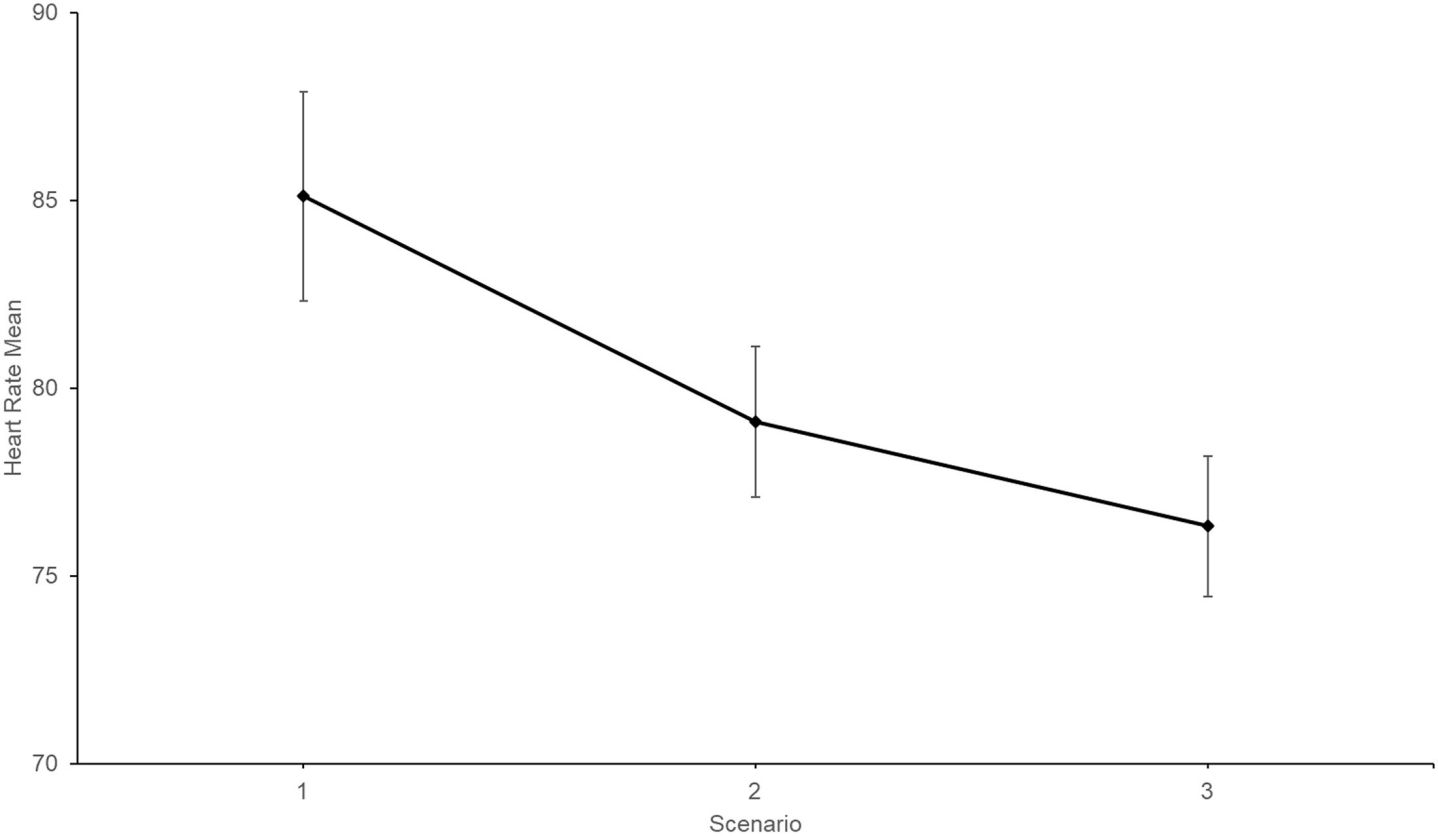
Pre-speech heart rate Time 1 mean scores for each scenario. Time 1 = 00:00 minutes. Error bars represent standard errors. (*N* = 19).

For heart rate, a repeated measures ANOVA confirmed habituation between some scenarios, *F* (2,36) = 383.28, *p* < .001, partial η^2^ = .37. Fig 8 shows the observed between-scenario heart rate trend. Pairwise comparisons are shown in Table 4.

## Discussion

The present study examined self-reported distress habituation during three brief repeated VR public speaking scenarios in a non-clinical adult sample. Within-speech repeated measures were utilised to gauge the efficacy of agent-based software in initiating public speaking distress and habituation. Distress was operationalised using SUDS for psychological distress, and heart rate for physiological arousal. SIAS mean score was indicative of a non-clinical community sample [31] and demonstrated good internal consistency. IPQ Total Score confirmed VR presence was achieved in relation to the criteria, which therefore indicates that participants experienced a sense of immersion and presence within the VRE scenarios [36].

As hypothesised, agent-based VRE elicited subjective distress during all of the utilised public speaking scenarios. Significant physiological arousal was also observed in heart rate data. These subjective distress and heart rate results support previous research [26, 30]. Furthermore, this study demonstrated that Virtual Orator software elicited significant levels of distress upon first view of the agent audience, prior to speech-performance cognitive load (i.e., cognition required for language planning and information processing during speech delivery [38]).

The second hypothesis, relating to within-scenario distress habituation, was not supported. While within-scenario habituation was evident during Scenario 1 and Scenario 3, results were not significant. Furthermore, Scenario 2 within-scenario distress increased. These results were not consistent with Finn, Sawyer [25], who reported significant within-scenario distress habituation during in vivo exposure. Since Finn, Sawyer [25] participants presented speeches to peers, audience familiarity could have enhanced habituation due to a reduced potential for negative evaluation and mutual empathy (audience members were also presenting speeches). In contrast, the present study utilised generic VR venues and agent audiences, ensuring that participants were blind to content pre-exposure. Additionally, Finn, Sawyer [25] habituation data was based on post-speech rather than within-scenario measures, with assessment speech scenarios being administered several weeks apart.

It remains unclear why Scenario 2 distress trended upward in the present study. At the 3-minute Scenario 2 milestone (i.e., Time 5), mean SUDS scores were not significantly different from Scenario 1 commencement (i.e., Time 1). It is possible that during Scenario 2, emotional processing may have not yet been achieved [23], or that distress tolerance diminished. Distress tolerance has been negatively correlated with anxiety symptomology [39]. As such, heightened awareness of new symptoms (e.g., sweaty palms) could have increased distress. Importantly, the increase in self-report distress within Scenario 2 indicated that three brief scenarios were required to achieve habituation (speech duration was not relevant). Therefore, single and dual speech VRE designs could inadvertently exacerbate FOPS.

The third hypothesis, concerning between-scenario distress habituation, was partially supported. Significant between-scenario public speaking distress habituation was observed between Scenario 1 and Scenario 2, and Scenario 1 and Scenario 3, however not between Scenario 2 and Scenario 3. One possible explanation for this is that distress escalation during Scenario 2 diminished participants’ confidence, thus increasing Scenario 3 pre-speech anxiety [40].

Between-scenario results demonstrated that randomisation of the scenarios (i.e., 8–46 agents) did not affect within-*session* distress habituation (i.e., from commencement of Scenario 1 to Scenario 3 conclusion). Participant’s distress reduced over the three scenarios even though scenario intensity was not increased systematically. Therefore, the present results do not support the rationale argued by Kozasa and Leite [29], and Harris, Kemmerling [26] that distress stability, despite increased scenarios difficulty, is an indication of distress habituation. However, it is still possible that participants in the Kozasa and Leite [29], and Harris, Kemmerling [26] studies experienced environmental-familiarity (i.e., venue and/or audience), which assisted with anxiety regulation (i.e., anxiety stability). Importantly, outcomes from this randomised, repeated-measures study demonstrated that agent-based VRE was not susceptible to environmental-familiarity. Furthermore, in contrast to commonly used post-exposure distress scores, the repeated-measures distress data utilised in the present study eliminated the need for distress variability assumptions.

### Present research Findings and future Recommendations

This pilot study contributes to existing public speaking VRE research by demonstrating that distress habituation is neither uniform nor systematic. Repetition of presentations was essential in achieving sustained distress habituation. While this finding requires replication, it has implications for research, as well as educational institutions. Single speech exposure could exacerbate FOPS by confirming participants’ anticipated anxiety without achieving habituation. Thus, participants may not experience sufficient positive emotion from a single scenario. This is an important consideration given that FOPS research commonly utilises single scenarios. Similarly, student academic public speaking tasks can be infrequent and limited to single speeches. Therefore, VRE could offer a cost-effective solution for schools to facilitate brief repeated, public speaking practice to build speakers’ confidence.

The present study also demonstrated that FOPS repeated distress measures and graded scenario randomisation are easily incorporated into VRE research designs. Measuring within-speech distress during performances provides a more detailed representation of distress variability than post-speech assessments; within-speech data facilitates better insight concerning emotional regulation and habituation. This benefits individual studies, as well as future literature comparisons. Understanding how habituation patterns differ between clinical and non-clinical populations could help improve FOPS detection, clinical diagnosis and treatment. As such, future VRE FOPS research should continue to utilise a repeated-measures design. Repeated measures and randomisation methodologies should also be considered for other VRE applications.

### Limitations

A number of limitations need to be considered. First, the small sample size, while not unusual for FOPS research, is more prone to type I error. Second, SUDS measures during VRE were cued by a manual call bell within the physical room. This could impact several IPQ question responses related to awareness of the real environment (i.e., physical room). To minimise this distraction, future designs should incorporate a within-software SUDS cue signal. Nevertheless, this was also considered a strength of the research design, as explained above. In addition, the current design did not consider the reduced timeframes for consolidation of emotional learning (i.e., only three minutes of rest were provided between scenarios). Thus, future within-scenario distress variability utilising brief, successive FOPS scenarios should be compared with extended VRE sessions; ideally single speeches separated by days to allow consolidation during sleep.

Furthermore, the current study did not evaluate the transfer of brief VRE exposure to real-world scenarios, or the long-term maintenance of FOPS habituation. Lastly, the inclusion of Toastmasters participants (*n* = 5) warrants a mention. It is important to consider that membership of Toastmasters does not necessarily represent a specific level of public speaking fear or competence (i.e., high or low). In other words, variability of public speaking skills and FOPS are to be expected, as per the general population. Further to this, clinical levels of social phobia were an exclusion criteria. However, sub-clinical levels of FOPS are common in the general population and also associated with distress and adverse consequences for work and vocational performance [41].

### Conclusion

The present VRE public speaking study demonstrated that rapidly successive FOPS scenarios were capable of eliciting self-reported distress within a non-clinical adult sample. Distress was easier to initiate than habituation, with three successive speeches required to sustain distress reduction. Replication of this finding will be valuable given that less frequent brief exposures could exacerbate FOPS. The present design also demonstrated that public speaking distress was elicited upon initial view of agent-based VR scenarios, prior to speech commencement. VRE has potential to enhance FOPS detection and therapy, making it cheaper, more accessible and more effective. To achieve this, VRE public speaking research should consider the value of a repeated-measures design to map distress variability and habituation; a methodology equally valuable for other VRE clinical research.
